# Enhancing NAD^+^ salvage metabolism is neuroprotective in a PINK1 model of Parkinson's disease

**DOI:** 10.1242/bio.022186

**Published:** 2016-12-23

**Authors:** Susann Lehmann, Samantha H. Y. Loh, L. Miguel Martins

**Affiliations:** MRC Toxicology Unit, Lancaster Road, Leicester LE1 9HN, UK

**Keywords:** *Drosophila*, Mitochondria, NAD^+^, NAM, Niacin, Nucleotide metabolism, Parkinson's disease, PARP, PINK1

## Abstract

Familial forms of Parkinson's disease (PD) caused by mutations in *PINK1* are linked to mitochondrial impairment. Defective mitochondria are also found in *Drosophila* models of PD with *pink1* mutations. The co-enzyme nicotinamide adenine dinucleotide (NAD^+^) is essential for both generating energy in mitochondria and nuclear DNA repair through NAD^+^-consuming poly(ADP-ribose) polymerases (PARPs). We found alterations in NAD^+^ salvage metabolism in *Drosophila pink1* mutants and showed that a diet supplemented with the NAD^+^ precursor nicotinamide rescued mitochondrial defects and protected neurons from degeneration. Additionally, a mutation of *Parp* improved mitochondrial function and was neuroprotective in the *pink1* mutants. We conclude that enhancing the availability of NAD^+^ by either the use of a diet supplemented with NAD^+^ precursors or the inhibition of NAD^+^-dependent enzymes, such as PARPs, which compete with mitochondria for NAD^+^, is a viable approach to preventing neurotoxicity associated with mitochondrial defects.

## Abstract (precis)

**Summary:** Dietary supplementation with an NAD^+^ salvage metabolite or decreasing Parp activity suppress mitochondrial dysfunction and is neuroprotective in a PINK1 model of Parkinson's disease.

## INTRODUCTION

Parkinson's disease (PD) is an age-associated neurodegenerative disorder characterised by the specific loss of dopaminergic neurons in the substantia nigra pars compacta of the brain. Most cases of PD are sporadic, but 5–10% of cases are inherited through PD-related genes (Bonifati, 2007; Gasser, 2009). Mitochondria have a crucial role in supplying energy to the brain, and their deterioration has long been associated with neurodegenerative states such as PD (reviewed in de Castro et al., 2010). Mitochondrial health is maintained by quality control (QC) mechanisms, such as the selective degradation of defective organelles by mitophagy, a form of autophagy. Mitophagy involves accumulation of the kinase PINK1 in the outer mitochondrial membrane of defective mitochondria, where it cooperates with the E3 ligase Parkin and FBXO7, an E3 ligase adaptor, to promote their autophagic degradation (reviewed in Celardo et al., 2014). Mutations in *PINK1*, *PARKIN* and *FBXO7* have been identified in families with autosomal recessive early-onset PD (Di Fonzo et al., 2009; Kitada et al., 1998; Valente et al., 2004). This suggests that defects in mitophagy might have a causative role in PD. Defects in mitophagy caused by mutations in *Pink1* lead to disruption of mitochondrial bioenergetics and alterations in the redox state of the complex I substrate nicotinamide adenine dinucleotide (NAD^+^) (Gandhi et al., 2009; Tufi et al., 2014). NAD^+^ also acts as co-enzyme for poly(ADP-ribose) polymerases (PARPs), which are major NAD^+^-consuming enzymes involved in nuclear DNA repair of healthy cells.

PARP over-activation has been associated with dopaminergic neuron toxicity and atrophy (Kim et al., 2013; Lee et al., 2013), as well as disruption of the mitochondrial ultrastructure (Virág and Szabó, 2002). In models of mitochondrial dysfunction associated with the loss of Parkin or FBXO7 function, it has been recently reported that decreasing the activity of PARPs, or increasing the bioavailability of NAD^+^ through dietary supplementation, can improve mitochondrial function and prevent neurodegeneration (Delgado-Camprubi et al., 2016; Lehmann et al., 2016).

The fruit fly *Drosophila melanogaster* is a powerful animal model for studying the mechanisms of neurodegeneration in PD. It is also an excellent *in vivo* system to test potentially neuroprotective compounds (reviewed in Lu and Vogel, 2009). *Drosophila pink1* mutants show mitochondrial dysfunction, resulting in the degeneration of muscle tissues, which causes a defective (crushed) thorax phenotype (Clark et al., 2006; Park et al., 2006). They also show a selective, age-dependent loss of the protocerebral posterior lateral 1 cluster of dopaminergic neurons (Park et al., 2006).

Here, we describe that *pink1* mutant flies have decreased levels of NAD^+^ metabolites. We demonstrated that a diet supplemented with an NAD^+^ precursor vitamin, nicotinamide (NAM), and the genetic suppression of *Parp*, a NAD^+^-consuming enzyme, improves mitochondrial function and prevents neurodegeneration in *pink1* mutant flies.

## RESULTS

### An NAD^+^-supplemented diet suppresses both mitochondrial defects and neurodegeneration in *pink1* mutant flies

**Fig. 1.**
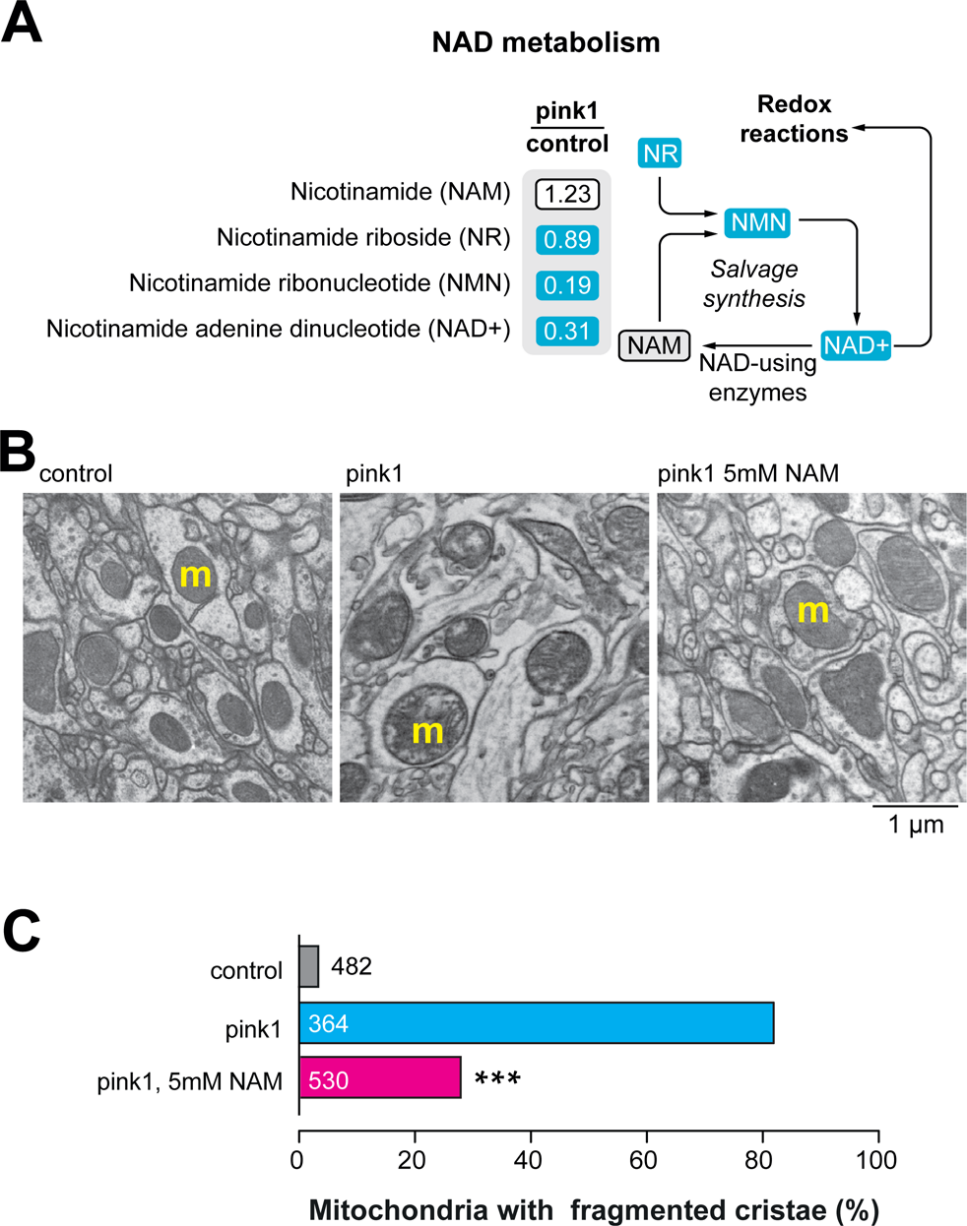
**Dietary supplementation with NAM suppresses mitochondrial defects in *pink1* mutant brains.** (A) Loss of *pink1* decreases NAD^+^ metabolite levels. Blue corresponds to metabolites that are significantly downregulated (*P*<0.05) compared to control. Statistical significance was determined using Welch's two-sample *t*-test (*n*=8). (B,C) An NAM-supplemented (5 mM) diet suppresses mitochondrial cristae fragmentation in *pink1* mutant brains. (B) Ultrastructural analysis of the adult brains of *pink1* mutants, showing mitochondria with fragmented cristae in neuropiles (m, mitochondria). Representative TEM micrographs of the indicated genotypes and treatments are shown. (C) Percentages of neuropile mitochondria exhibiting fragmented cristae normalised to the area are presented (asterisks, two-tailed chi-square test, 95% confidence intervals). Datasets labelled control and pink1 are also used in Fig. 3E. Genotypes: control: *w^1118^*, pink1: *pink1^B9^*.

**Fig. 2.**
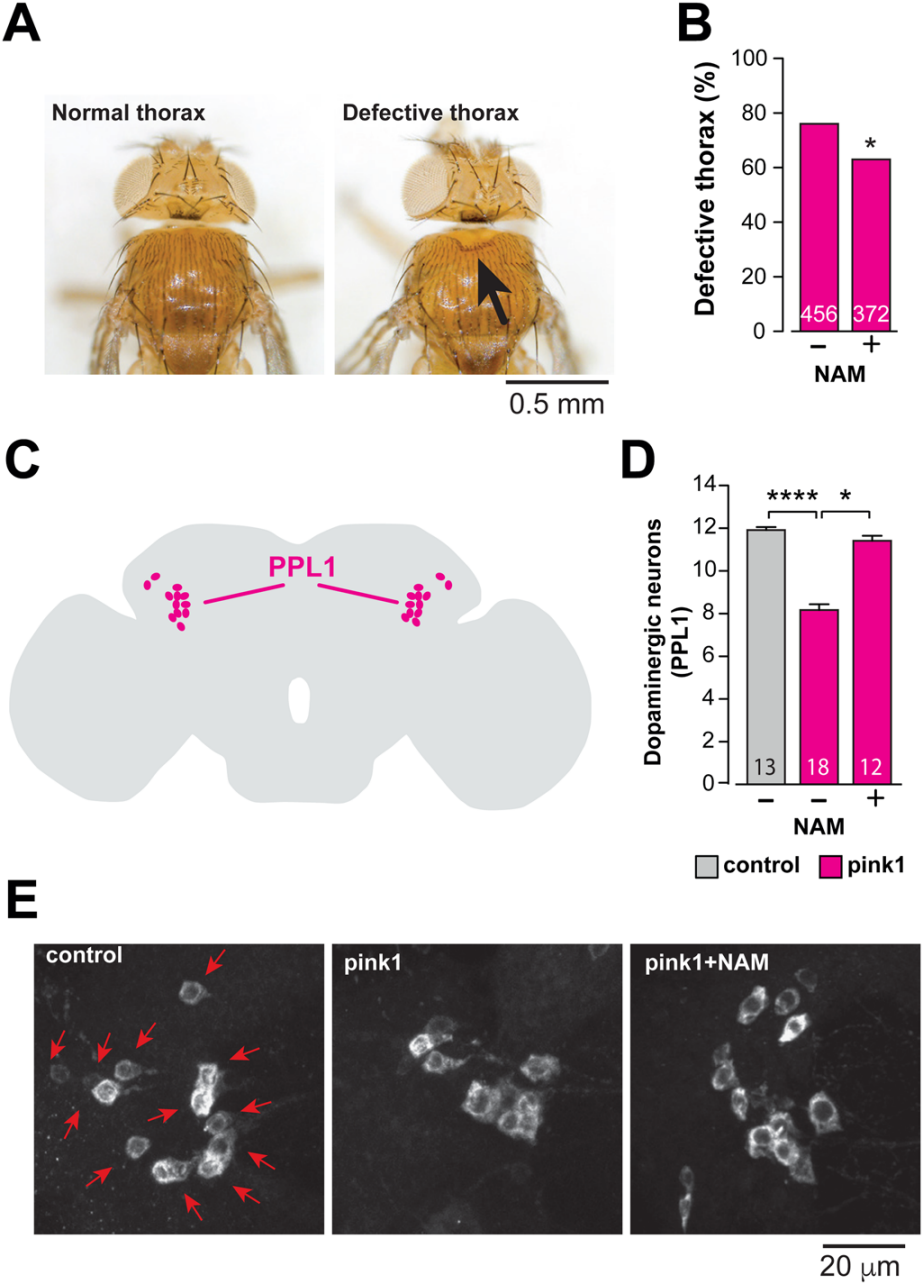
**An NAM-enhanced diet suppresses neurodegeneration in *pink1* mutants.** (A,B) Dietary supplementation with NAM (5 mM) rescues the thoracic defects of *pink1* mutants. (A) Representative images of normal and defective thorax in *pink1* mutants, the arrow points to a thoracic defect. (B) Quantification of the thoracic defects of *pink1* mutants fed on a normal or 5 mM NAM-supplemented diet (asterisks, two-tailed chi-square test, 95% confidence intervals). (C-E) An NAM-enhanced diet rescues the loss of dopaminergic neurons in the PPL1 cluster of *pink1* mutant flies. (C) Schematic diagram of an adult fly brain in the sagittal orientation, with PPL1 cluster neurons coloured magenta. (D) Quantification of PPL1 cluster neurons (mean± s.d.; asterisks, two-tailed unpaired *t*-test) and (E) representative images of anti-tyrosine hydroxylase staining showing cell bodies (red arrows) of PPL1 neurons of the indicated genotypes and treatments. Genotypes: control: *w^1118^*, pink1: *pink1^B9^*.

NAD^+^ metabolism plays a crucial role in PD pathogenesis in a number of PD-associated disease models that present with mitochondrial defects (Delgado-Camprubi et al., 2016; Lehmann et al., 2016). We have previously found low NAD^+^ levels in *parkin* mutants (Lehmann et al., 2016), a downstream effector of *pink1* (Clark et al., 2006; Park et al., 2006). To determine whether the loss of *pink1* directly affects NAD^+^ metabolism, we analysed the levels of NAD^+^ metabolites in *pink1* mutants by global metabolic profiling. We detected significant reductions in the level of NAD^+^, as well as the NAD^+^ salvage metabolite nicotinamide ribonucleotide (NMN) and NAD^+^ precursor nicotinamide riboside (NR) (Fig. 1A). In the *pink1* mutants, NAD^+^ levels were decreased, we therefore assessed whether enhancing the NAD^+^ salvage synthesis using NAM could prevent the mitochondrial defects in these mutants. Degeneration of the indirect flight muscle in *pink1* mutants is associated with the fragmentation of the mitochondrial cristae in these tissues (Clark et al., 2006; Park et al., 2006; Tufi et al., 2014). We also detected mitochondrial cristae fragmentation in the neuropiles of the adult brains of *pink1* mutants (Fig. 1B). Maintenance of the *pink1* mutants on a diet supplemented with NAM resulted in reduced numbers of mitochondria with fragmented cristae (Fig. 1B,C). In addition, maintaining *pink1* mutants in an NAM-supplemented diet also reduced the number of flies with a defective thorax (Fig. 2A,B) and prevented the loss of dopaminergic neurons (Fig. 2C-E). Together these results indicate that dietary supplementation with the NAD^+^ precursor NAM improves mitochondrial function and is neuroprotective in *pink1* mutants.

### Mutation of *Parp* rescues mitochondrial dysfunction of *pink1* mutants

Depletion of cellular NAD^+^ levels has been linked to enhanced oxidative stress, which causes activation of the NAD^+^-consuming PARP enzymes (Virág et al., 1998). We have previously shown that *Drosophila parkin* mutants have increased levels of the oxidative stress markers methionine sulfoxide and homocysteine, a marker of oxidative stress and a metabolite that generates reactive oxygen species upon auto-oxidation, respectively (Lehmann et al., 2016).

**Fig. 3.**
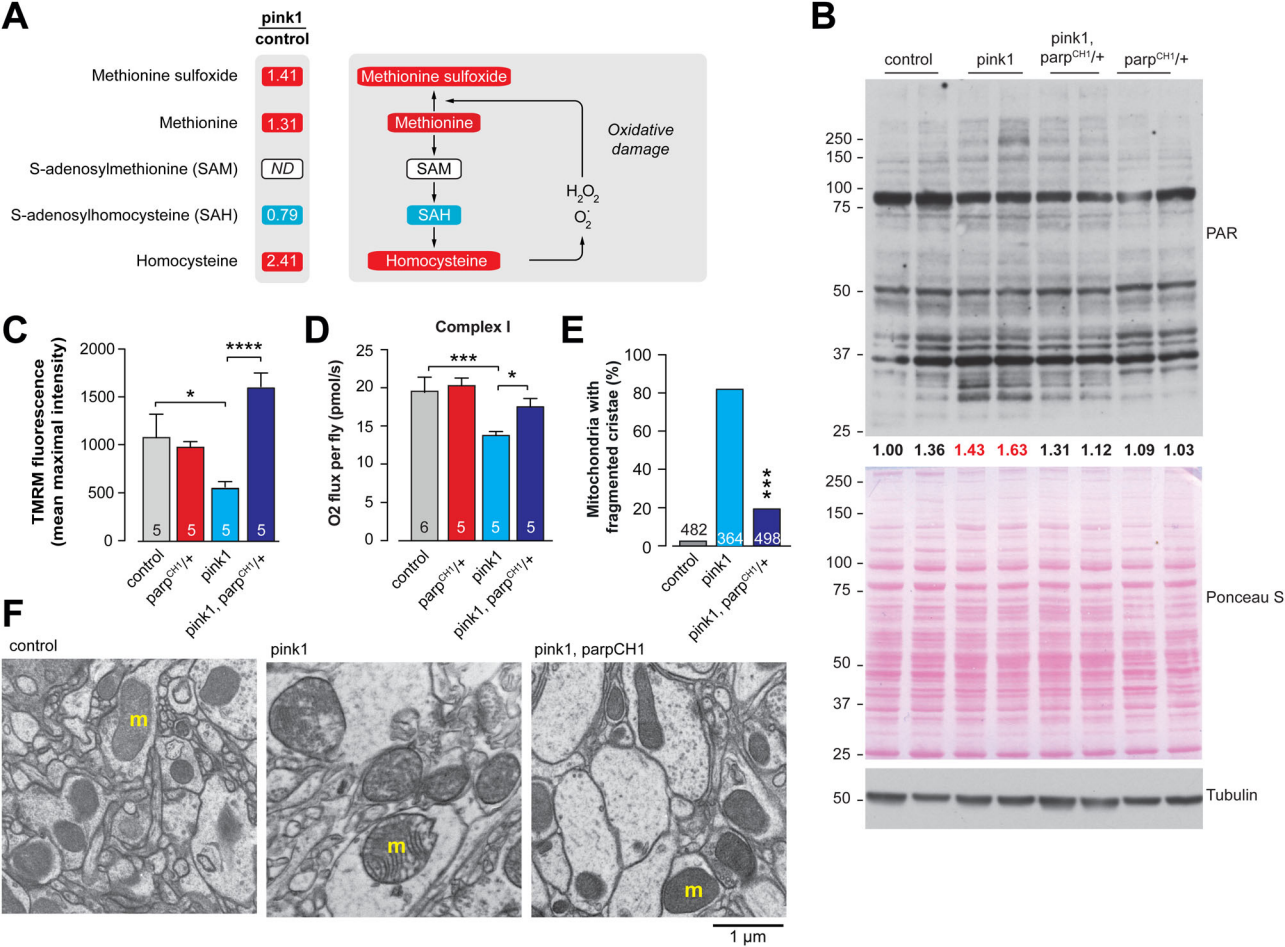
***Parp* mutation rescues mitochondrial function in *pink1* mutants.** (A) The levels of oxidative stress-related metabolites are increased in *pink1* mutants. The metabolites indicated in red or blue are significantly upregulated or downregulated, respectively, compared to control (*P*<0.05). ND corresponds to a metabolite below detection threshold. The statistical significance for fold-changes was determined using Welch's two-sample *t*-test (*n*=8). (B) Protein PARylation is increased in *pink1* mutants, and this increase is attenuated in *pink1, Parp^CH1^/+* double mutants. Whole-fly lysates were analysed using the indicated antibodies. Tubulin was used as a loading control. Ponceau S staining was used to assess total protein load. Ratios of signal intensity between total PAR and total protein load (Ponceau S) are presented. Two biological replicates are shown for each genotype. Red text indicates ratios of samples with high level of PARylation. (C) *Parp* mutation protects against the loss of Δψm in *pink1* mutants (mean±s.d.; asterisks, one-way ANOVA with Bonferroni's multiple comparison test). (D) *Parp* mutation increases complex I-mediated respiration in *pink1* mutants (mean±s.d.; asterisks, one-way ANOVA with Bonferroni's multiple comparison test). Datasets in C and D labelled ‘control’ and ‘*Parp^CH1^/+*’ have been previously published in Lehmann et al. (2016), as data from these genotypes were obtained as a single experimental set before statistical analysis. (E,F) *Parp* mutation rescues mitochondrial cristae fragmentation in *pink1* mutant brains. (E) Percentages of neuropile mitochondria exhibiting fragmented cristae normalised to the area are presented for the indicated genotypes (asterisks, two-tailed chi-square test, 95% confidence intervals). Datasets labelled control and pink1 are also used in Fig. 1C. (F) Representative TEM micrographs of the indicated genotypes are shown (m, mitochondria). Genotypes: control: *w^1118^*, pink1: *pink1^B9^*, pink1, parp^CH1^/+: *pink1^B9^, Parp^CH1^*/*+.*

Metabolic profiling performed in this study revealed increased levels of these same oxidative markers, as well as of methionine (Fig. 3A), and increased protein PARylation (a post-translational protein modification carried out by PARPs using NAD^+^ as a substrate) in the *pink1* mutants (Fig. 3B). Therefore, we assessed whether the depletion of NAD^+^ observed in the *pink1* mutants was associated with the enhanced activity of PARP. To address this, we examined whether a loss-of-function mutation in the *Parp* gene in *Parp^CH1^/+* flies (Ong et al., 2013; Tulin et al., 2002; Zhang and Spradling, 1994) could reverse the mitochondrial impairment of *pink1* mutants. We demonstrated that the *Parp* gene mutation attenuated the enhanced protein PARylation in the *pink1* mutants (Fig. 3B), indicating that this mutation decreased the overall PARP activity. Next, we examined whether the *Parp* mutation could improve the mitochondrial health of the *pink1* mutants. We demonstrated that the *Parp* mutation suppressed the loss of Δψm (Fig. 3C) and restored complex I-mediated respiration in the *pink1* mutants (Fig. 3D). In addition, *Parp* mutation also reduced the defects in mitochondrial morphology in the adult brains of the *pink1* mutants (Fig. 3E,F). Taken together, these results indicate that reduction in the activity of an NAD^+^-consuming enzyme, such as Parp, results in improved mitochondrial function in *pink1* mutants.

### *Parp* mutation is neuroprotective in *pink1* mutant flies

**Fig. 4.**
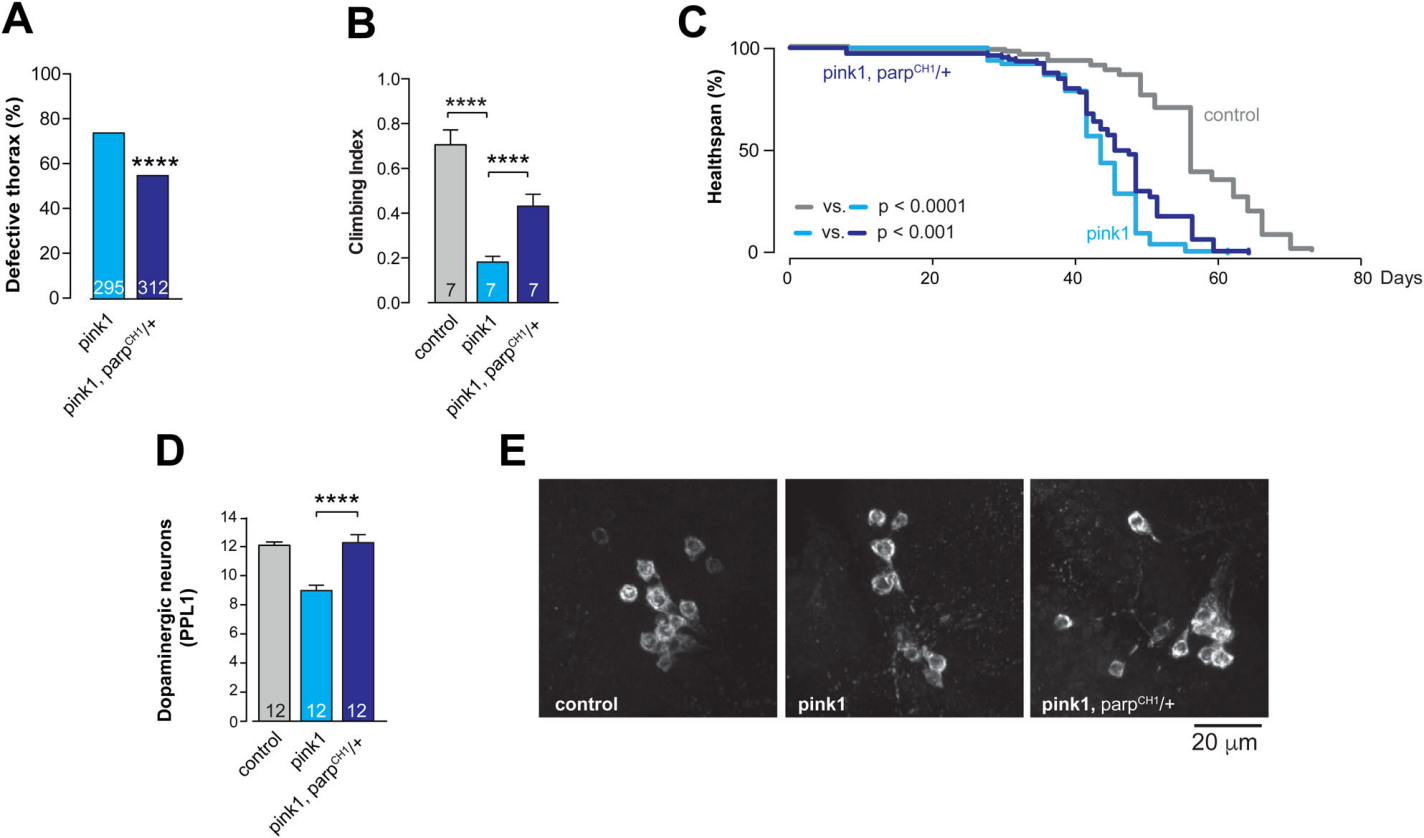
***Parp* mutation rescues *pink1* mutant phenotype.** (A) *Parp* mutation rescues the thoracic defect (asterisks, two-tailed chi-square test, 95% confidence intervals), (B) climbing ability (mean±s.d.; asterisks, two-tailed unpaired *t*-test) and (C) increases survival of *pink1* mutants (*n*=130 for control, *n*=114 for *pink1*, and *n*=106 for *pink1*, *Parp^CH1^/+*; asterisks, log-rank Mantel-Cox test). (D) *Parp* mutation rescues the loss of dopaminergic neurons in the PPL1 cluster of *pink1* mutant flies (mean±s.e.m.; asterisks, one-way ANOVA with Bonferroni's multiple comparison test). (E) Representative images of anti-tyrosine hydroxylase-stained PPL1 cluster neurons are shown for the indicated genotypes. Genotypes: control: *w^1118^*, pink1: *pink1^B9^*, pink1, parp^CH1^/+: *pink1^B9^, Parp^CH1^*/*+.* Dataset in D labelled ‘control’ has been previously published in Lehmann et al. (2016), as data were obtained as a single experimental set before statistical analysis.

Next, we examined the effect of the *Parp* mutation on the neurodegenerative phenotypes of the *pink1* mutants by comparing the phenotype of the *pink1* flies to that of *pink1, Parp^CH1^/+* double mutants. We determined that the mutation of *Parp* was sufficient to reduce the thoracic indentation (Fig. 4A) and to improve the locomotive defects (Fig. 4B) of the *pink1* mutants. Moreover, *Parp* mutation increased the lifespan of the *pink*1 mutants (Fig. 4C). In addition, it rescued the loss of the PPL1 clusters of dopaminergic neurons in the *pink1* mutants (Fig. 4D,E). Collectively, these results indicate that the *Parp* gene mutation suppresses mitochondrial dysfunction and is neuroprotective in *pink1* mutants.

## DISCUSSION

Disruption of mitochondrial function is a key hallmark of PD (reviewed in Lehmann and Martins, 2013). Cells such as neurons have several QC systems in place, which act at the molecular, organellar and cellular levels to respond to mitochondrial defects (reviewed in de Castro et al., 2010). Defects in Pink1 and its downstream effectors, Parkin or Fbxo7, compromise mitophagy, a mechanism of organellar QC. Loss-of-function mutations in any of these three genes cause familial PD and result in the accumulation of defective mitochondria, leading to cellular toxicity, partly through the generation of toxic ROS. Pink1 is the key initiator of mitophagy, and its impairment affects mitochondrial bioenergetics and alters the redox state of the complex I substrate NAD^+^. Here, we have demonstrated the neuroprotective potential of NAM, a form of vitamin B3, and of the genetic suppression of Parp, an NAD^+^-consuming enzyme, in *pink1* mutant flies. Other studies using fly models have also shown that vitamin-based dietary interventions suppress mitochondrial dysfunction and block neurodegeneration *in vivo* (Lehmann et al., 2016; Tufi et al., 2014; Vos et al., 2012). In addition, a high level of dietary niacin, another form of vitamin B3, has also been reported to confer a reduced risk of developing PD (Fall et al., 1999; Hellenbrand et al., 1996). Altogether, these studies have demonstrated the therapeutic potential of a vitamin-enriched diet in a genetic animal model of PD associated with mitochondrial dysfunction. However, based on the available body of evidence, we reason that although vitamin interventions might delay or prevent neurodegeneration in diseases associated with mitochondrial defects, such as PD, they cannot be considered potential ‘cures’ because they cannot reverse the loss of specific populations of neurons that are absent at the time of diagnosis.

We observed the upregulation of markers of oxidative stress in the *pink1* mutants. Oxidative damage of DNA molecules activates PARP enzymes and plays an important role in PD (Zuo and Motherwell, 2013). The enhanced activation of PARPs in this context can result in depletion of the cellular stores of metabolites required for mitochondrial function, such as NAD^+^ and ATP, the cellular energy currency, thereby decreasing the availability of these metabolites for other essential cellular processes.

Studies of the human PARP-1 gene have identified several polymorphisms linked to susceptibility to various diseases (Kato et al., 2000; Pascual et al., 2003). For example, the active-site polymorphism T2444C has been reported to decrease PARP-1 activity by approximately 40% (Wang et al., 2007). The data presented here, along with those of two other recent reports (Delgado-Camprubi et al., 2016; Lehmann et al., 2016), indicate that the inhibition of PARPs could be a viable strategy to protect neurons from the neurotoxic consequences of mutations in genes encoding mitophagy components, such as *PINK1*, *PARKIN* and *FBXO7*. Therefore, it would be interesting to examine whether PD patients carrying mutations in these genes, as well as potential loss-of-function polymorphisms in PARPs, display less severe disease symptoms and have a higher life expectancy.

We conclude that a potential therapeutic approach aimed at increasing the NAD^+^ level by using vitamin precursors or inhibiting PARP activity could be useful for the treatment of several mitochondrial dysfunction-associated forms of PD. As PARP inhibitors are already in use in clinical studies of cancer and in stroke treatment, this work expands the application of this potential therapeutic agent to treatment of a disease that, thus far, has no cure.

## MATERIALS AND METHODS

### Genetics and *Drosophila* strains

Fly stocks and crosses were maintained on standard cornmeal agar media at 25°C. The strains used were *pink1^B9^* (a kind gift from A. Whitworth, MRC, Centre for Developmental and Biomedical Genetics, University of Sheffield, Sheffield, UK), *Parp^CH1^* (a kind gift from V. Corces, Department of Biology, Emory University, Atlanta, GA, USA and A. Tulin, Fox Chase Cancer Centre, Philadelphia, PA, USA) and *w^1118^* (Bloomington Stock Centre). All experiments on adult flies were performed using males.

### Metabolic profiling

Global metabolic profiles were obtained from 3-day-old flies using the Metabolon Platform (Metabolon Inc., NC, USA) as previously described (Tufi et al., 2014). Essentially, each sample consisted of eight biological replicates (100 flies per replicate). The sample preparation process was carried out using an automated MicroLab STAR^®^ system (Hamilton Robotics, Reno, NV, USA). For sample extraction, an 80% (v/v) methanol:water solution was used. Samples were then prepared for the appropriate analysis (either LC/MS or GC/MS). Compounds above the detection threshold were identified by comparison to library entries of purified standards or recurrent unknown entities present in Metabolon's proprietary LIMS system. Identification of known chemical entities was based on comparison to metabolomic library entries of purified standards.

### Dietary supplements

NAM-supplemented diets were prepared as previously described (Lehmann et al., 2016). Briefly, NAM was incorporated into the fly food at a final concentration of 5 mM. Crosses were set up on normal food, and transferred to NAM-containing food after two days. Larvae were treated with NAM throughout development. Adult flies were maintained on NAM-containing food throughout their lifespan, and they were transferred to vials with fresh food every two to three days.

### Microscopy-based assessment of mitochondrial function

Measurement of Δψm in brains of 3-day-old flies was performed using tetramethylrhodamine (TMRM) as previously described (Tufi et al., 2014). Briefly, fly brains were loaded with 40 nM TMRM in loading buffer (10 mM HEPES, pH 7.35, 156 mM NaCl, 3 mM KCl, 2 mM MgSO_4_, 1.25 mM KH_2_PO_4_, 2 mM CaCl_2_ and 10 mM glucose) for 40 min at room temperature, and the dye was present during the experiment. In this experiment, TMRM was used in the redistribution mode to assess Δψm, and therefore, a reduction in TMRM fluorescence represents mitochondrial depolarisation. Confocal images were obtained using a Zeiss 510 confocal microscope equipped with a 40x oil immersion objective. Illumination intensity was kept to a minimum (at 0.1-0.2% of laser output) to avoid phototoxicity, and the pinhole was set to give an optical slice of 2 µm. Fluorescence was quantified by exciting TMRM using the 565 nm laser and measured above 580 nm. Z-stacks of 5 fields of 300 μm^2^ each per brain were acquired, and the mean maximal fluorescence intensity was measured for each group.

### Defective thorax analysis

Visual assessment of thoracic indentations (defective thorax) was performed essentially as a binary assay, as previously described (Lehmann et al., 2016). First, we determined whether each fly had a defective thorax, and second, we used the chi-square test to determine whether the degree (percentage) of the crushed thorax phenotypes in the populations under analysis is significantly different.

### Analysis of dopaminergic neurons

Brains from 20-day-old flies were dissected and stained using anti-tyrosine hydroxylase (Immunostar, WI, USA) as previously described (Whitworth et al., 2005). The brain samples were placed in PBS+0.1% Triton in a coverslip clamp chamber (ALA Scientific Instruments Inc., NY, USA), positioned using a harp-like apparatus made of platinum wire and nylon string and imaged by confocal microscopy. Tyrosine hydroxylase-positive PPL1 cluster neurons were counted per brain hemisphere. Data acquired for the assessment of each genotype were obtained as a single experimental dataset before statistical analysis.

### Protein extraction and western blotting

Protein extracts from whole flies were prepared by grinding the flies in lysis buffer [100 mM KCl, 20 mM HEPES, pH 7.5, 5% (v/v) glycerol, 10 mM EDTA, 0.1% (v/v) Triton X-100, 10 mM DTT, 1 μg/ml leupeptin, 1 μg/ml antipain, 1 μg/ml chymostatin, and 1 μg/ml pepstatin] as previously described (Lehmann et al., 2016). The suspensions were cleared by centrifugation at 21,000* **g*** for 10 min at 4°C, and the protein concentrations of the supernatants were determined by Bradford assay (Bio-Rad). All supernatants were mixed with 4× LDS loading buffer. For SDS-PAGE, equivalent amounts of proteins were resolved on 4-12% NuPAGE Precast Gels (Invitrogen, MA, USA) and transferred onto nitrocellulose membranes (Millipore, MA, USA) for incubation with α-PAR or to PVDF membranes (Millipore, MA, USA) for incubation with α-Tubulin. The membranes were blocked with TBS (0.15 M NaCl and 10 mM Tris-HCl, pH 7.5) containing 5% (w/v) dried non-fat milk for 1 h at room temperature and were then probed with the indicated primary antibody, followed by incubation with the appropriate HRP-conjugated secondary antibody. Antibody complexes were visualised using Pierce's enhanced chemiluminescence system (ECL). The levels of total PAR were calculated as ratio to the total protein load, which was visualised by staining the membrane with Ponceau S [0.1% Ponceau S (w/v), 5% acetic acid (w/v)], and performing densitometry analysis with ImageJ software (http://imagej.nih.gov/ij/; provided in the public domain by the National Institutes of Health, Bethesda, MD, USA).

### Antibodies

The primary antibodies employed in this study included Tyrosine Hydroxylase (1:50, Immunostar, WI, USA), PAR (1:500, Trevigen, MD, USA, 4335-MC) and α-Tubulin (1:5000, Sigma, T6074).

### Respirometry

Mitochondrial respiration in 3-day-old flies was assayed at 37°C by high-resolution respirometry as previously described (Costa et al., 2013). OROBOROS Oxygraph DatLab software package (OROBOROS, Innsbruck, Austria) was used for data acquisition (2-s time intervals) and analysis, including calculation of the time derivative of the oxygen concentration and signal deconvolution dependent on the response time of the oxygen sensor, with correction for instrumental background oxygen flux. Respiration was assessed by homogenising two flies using a pestle in MiR05 respiration buffer (20 mM HEPES, 10 mM KH_2_PO_4_, 110 mM sucrose, 20 mM taurine, 60 mM K-lactobionate, 0.5 mM EGTA, 3 mM MgCl_2_, and 1g/l fatty acid-free BSA). Coupled state 3 respiration for complex I was assayed in MiR05 respiration buffer in the presence of 2 mM malate, 10 mM glutamate and 5 mM ADP.

### Climbing assay

Climbing assays were performed as previously described (Greene et al., 2003) using a counter-current apparatus equipped with six chambers. A total of 15 to 20 male 3-day-old flies were placed into the first chamber, tapped to the bottom, and then given 20 s to climb a distance of 10 cm. The flies that successfully climbed 10 cm or beyond within 20 s were then shifted to a new chamber, and both sets of flies were given another opportunity to climb the 10-cm distance. This procedure was repeated a total of five times. After five trials, the number of flies in each chamber was counted. A video demonstrating this technique can be found at https://youtu.be/vmR6s_WAXgc. The climbing index was measured using a weighted average approach with the following formula:

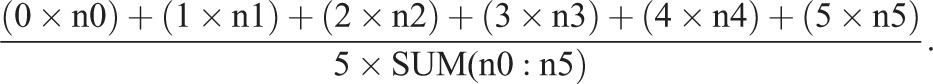


In this formula, n0 corresponds to the number of flies that failed the first trial, and n1 through n5 are the numbers of flies that successfully passed each successive trial. At least 100 flies were used for each genotype tested.

### Lifespan analysis

Lifespan analysis was conducted as previously described (Lehmann et al., 2016). Briefly, groups of 15 newly enclosed males of each genotype were placed into separate vials with food and maintained at 25°C. The flies were transferred to vials containing fresh food every two to three days, and the number of dead flies was recorded. The data are presented as Kaplan–Meier survival distributions, and significance was determined by the log-rank test.

### Electron microscopy

For transmission electron microscopy (TEM), adult fly brains were fixed overnight in 0.1 M sodium cacodylate buffer (pH 7.4) containing 2% paraformaldehyde, 2.5% glutaraldehyde and 0.1% Tween-20. Then, the samples were post-fixed for 1 h at room temperature in a solution containing 1% osmium tetroxide and 1% potassium ferrocyanide. After fixation, the samples were stained *en bloc* with 5% aqueous uranyl acetate overnight at room temperature; then, they were dehydrated via a series of ethanol washes and embedded in TAAB epoxy resin (TAAB Laboratories Equipment Ltd., Aldermaston, UK). Semi-thin sections were stained with Toluidine Blue, and areas of the sections were selected for ultramicrotomy. Ultrathin sections were stained with lead citrate and imaged using a MegaView 3 digital camera and iTEM software (Olympus Soft Imaging Solutions GmbH, Münster, Germany) with a Jeol 100-CXII electron microscope (Jeol UK Ltd., Welwyn Garden City, UK). Data acquired in the assessments of both the genetic and pharmacological rescue of the *pink1* mutants were obtained as a single experimental dataset before statistical analysis.

### Statistical analyses

Descriptive and inferential statistical analyses were performed using GraphPad Prism 6 (www.graphpad.com). The data are presented as the mean value, and the error bar indicates±s.d. or ±s.e.m. (as indicated). The number of biological replicates per experimental variable (*n*) is indicated in either the respective figure or figure legend. Parametric tests were used (performed using data obtained from pilot experiments) after confirming that the variables under analysis displayed a Gaussian distribution using the D'Agostino-Pearson test (computed using GraphPad Prism 6). Significance is indicated as ****P<0.0001, ***P<0.001, **P<0.01, and *P<0.05. For statistical analysis of metabolites in the flies, pairwise comparisons were performed using Welch's *t*-test. The q-value provides an estimate of the false discovery rate (FDR), according to Storey and Tibshirani (2003). The investigators gathering quantitative data on the biological samples were not blinded to the sample identities at the time of analysis. No specific randomisation strategies were employed when the biological replicates were assigned to the treatment groups.

### Digital image processing

Western blot images were acquired as uncompressed, bitmapped digital images (TIFF format). The images were processed using Adobe Photoshop CS5, employing established scientific imaging workflows (Wexler, 2008).

## Video abstract

A video abstract is available at: https://youtu.be/1e4oWI50IWg.

## References

[BIO022186C1] Bonifati V. (2007). Genetics of parkinsonism. *Parkinsonism Relat. Disord.* 13 Suppl. 3, S233-S241. 10.1016/S1353-8020(08)70008-7 18267242

[BIO022186C2] Celardo I., Martins L. M. and Gandhi S. (2014). Unravelling mitochondrial pathways to Parkinson's disease. *Br. J. Pharmacol.* 171, 1943-1957. 10.1111/bph.12433 24117181 PMC3976614

[BIO022186C3] Clark I. E., Dodson M. W., Jiang C., Cao J. H., Huh J. R., Seol J. H., Yoo S. J., Hay B. A. and Guo M. (2006). *Drosophila pink1* is required for mitochondrial function and interacts genetically with parkin. *Nature* 441, 1162-1166. 10.1038/nature04779 16672981

[BIO022186C4] Costa A. C., Loh S. H. Y. and Martins L. M. (2013). *Drosophila Trap1* protects against mitochondrial dysfunction in a PINK1/parkin model of Parkinson's disease. *Cell Death Dis.* 4, e467 10.1038/cddis.2012.205 23328674 PMC3563993

[BIO022186C5] de Castro I. P., Martins L. M. and Tufi R. (2010). Mitochondrial quality control and neurological disease: an emerging connection. *Expert Rev. Mol. Med.* 12, e12 10.1017/S1462399410001456 20398440 PMC2871738

[BIO022186C6] Delgado-Camprubi M., Esteras N., Plun-Favreau H. and Abramov A. Y. (2016). Deficiency of Parkinson's disease related gene Fbxo7 is associated with impaired mitochondrial metabolism by PARP activation. *Cell Death Differ*. in press 10.1038/cdd.2016.104 PMC5260490 27689878

[BIO022186C7] Di Fonzo A. D., Dekker M. C. J., Montagna P., Baruzzi A., Yonova E. H., Correia Guedes L., Szczerbinska A., Zhao T., Dubbel-Hulsman L. O., Wouters C. H. et al. (2009). FBXO7 mutations cause autosomal recessive, early-onset parkinsonian-pyramidal syndrome. *Neurology* 72, 240-245. 10.1212/01.wnl.0000338144.10967.2b 19038853

[BIO022186C8] Fall P.-A., Fredrikson M., Axelson O. and Granérus A.-K. (1999). Nutritional and occupational factors influencing the risk of Parkinson's disease: a case-control study in southeastern Sweden. *Mov. Disord.* 14, 28-37. 10.1002/1531-8257(199901)14:1<28::AID-MDS1007>3.0.CO;2-O 9918341

[BIO022186C9] Gandhi S., Wood-Kaczmar A., Yao Z., Plun-Favreau H., Deas E., Klupsch K., Downward J., Latchman D. S., Tabrizi S. J., Wood N. W. et al. (2009). PINK1-associated Parkinson's disease is caused by neuronal vulnerability to calcium-induced cell death. *Mol. Cell* 33, 627-638. 10.1016/j.molcel.2009.02.013 19285945 PMC2724101

[BIO022186C10] Gasser T. (2009). Molecular pathogenesis of Parkinson disease: insights from genetic studies. *Expert Rev. Mol. Med.* 11, e22 10.1017/S1462399409001148 19631006

[BIO022186C11] Greene J. C., Whitworth A. J., Kuo I., Andrews L. A., Feany M. B. and Pallanck L. J. (2003). Mitochondrial pathology and apoptotic muscle degeneration in *Drosophila parkin* mutants. *Proc. Natl. Acad. Sci. USA* 100, 4078-4083. 10.1073/pnas.0737556100 12642658 PMC153051

[BIO022186C12] Hellenbrand W., Boeing H., Robra B. P., Seidler A., Vieregge P., Nischan P., Joerg J., Oertel W. H., Schneider E. and Ulm G. (1996). Diet and Parkinson's disease. II: A possible role for the past intake of specific nutrients. Results from a self-administered food-frequency questionnaire in a case-control study. *Neurology* 47, 644-650. 10.1212/WNL.47.3.644 8797457

[BIO022186C13] Kato N., Morita H., Sugiyama T., Kurihara H., Tsubaki S., Nabika T., Kitamura K., Yamori Y. and Yazaki Y. (2000). Evaluation of the poly(ADP-ribose) polymerase gene in human stroke. *Atherosclerosis* 148, 345-352. 10.1016/S0021-9150(99)00284-1 10657571

[BIO022186C14] Kim T. W., Cho H. M., Choi S. Y., Suguira Y., Hayasaka T., Setou M., Koh H. C., Hwang E. M., Park J. Y., Kang S. J. et al. (2013). (ADP-ribose) polymerase 1 and AMP-activated protein kinase mediate progressive dopaminergic neuronal degeneration in a mouse model of Parkinson's disease. *Cell Death Dis.* 4, e919 10.1038/cddis.2013.447 24232095 PMC3847323

[BIO022186C15] Kitada T., Asakawa S., Hattori N., Matsumine H., Yamamura Y., Minoshima S., Yokochi M., Mizuno Y. and Shimizu N. (1998). Mutations in the parkin gene cause autosomal recessive juvenile parkinsonism. *Nature* 392, 605-608. 10.1038/33416 9560156

[BIO022186C16] Lee Y., Karuppagounder S. S., Shin J.-H., Lee Y.-I., Ko H. S., Swing D., Jiang H., Kang S.-U., Lee B. D., Kang H. C. et al. (2013). Parthanatos mediates AIMP2-activated age-dependent dopaminergic neuronal loss. *Nat. Neurosci.* 16, 1392-1400. 10.1038/nn.3500 23974709 PMC3785563

[BIO022186C17] Lehmann S. and Martins L. M. (2013). Insights into mitochondrial quality control pathways and Parkinson's disease. *J. Mol. Med. (Berl.)* 91, 665-671. 10.1007/s00109-013-1044-y 23644494

[BIO022186C18] Lehmann S., Costa A. C., Celardo I., Loh S. H. Y. and Martins L. M. (2016). Parp mutations protect against mitochondrial dysfunction and neurodegeneration in a PARKIN model of Parkinson's disease. *Cell Death Dis.* 7, e2166 10.1038/cddis.2016.72 27031963 PMC4823968

[BIO022186C19] Lu B. and Vogel H. (2009). *Drosophila* models of neurodegenerative diseases. *Annu. Rev. Pathol.* 4, 315-342. 10.1146/annurev.pathol.3.121806.151529 18842101 PMC3045805

[BIO022186C20] Ong C.-T., Van Bortle K., Ramos E. and Corces V. G. (2013). Poly(ADP-ribosyl)ation regulates insulator function and intrachromosomal interactions in *Drosophila*. *Cell* 155, 148-159. 10.1016/j.cell.2013.08.052 24055367 PMC3816015

[BIO022186C21] Park J., Lee S. B., Lee S., Kim Y., Song S., Kim S., Bae E., Kim J., Shong M., Kim J.-M., Chung J (2006). Mitochondrial dysfunction in *Drosophila* PINK1 mutants is complemented by parkin. *Nature* 441, 1157-1161. 10.1038/nature04788 16672980

[BIO022186C22] Pascual M., López-Nevot M. A., Cáliz R., Ferrer M. A., Balsa A., Pascual-Salcedo D. and Martín J. (2003). A poly(ADP-ribose) polymerase haplotype spanning the promoter region confers susceptibility to rheumatoid arthritis. *Arthritis. Rheum.* 48, 638-641. 10.1002/art.10864 12632415

[BIO022186C23] Storey J. D. and Tibshirani R. (2003). Statistical significance for genomewide studies. *Proc. Natl. Acad. Sci. USA* 100, 9440-9445. 10.1073/pnas.1530509100 12883005 PMC170937

[BIO022186C24] Tufi R., Gandhi S., de Castro I. P., Lehmann S., Angelova P. R., Dinsdale D., Deas E., Plun-Favreau H., Nicotera P., Abramov A. Y. et al. (2014). Enhancing nucleotide metabolism protects against mitochondrial dysfunction and neurodegeneration in a PINK1 model of Parkinson's disease. *Nat. Cell Biol.* 16, 157-166. 10.1038/ncb2901 24441527 PMC4199097

[BIO022186C25] Tulin A., Stewart D. and Spradling A. C. (2002). The *Drosophila* heterochromatic gene encoding poly(ADP-ribose) polymerase (PARP) is required to modulate chromatin structure during development. *Genes Dev.* 16, 2108-2119. 10.1101/gad.1003902 12183365 PMC186441

[BIO022186C26] Valente E. M., Abou-Sleiman P. M., Caputo V., Muqit M. M., Harvey K., Gispert S., Ali Z., Del Turco D., Bentivoglio A. R., Healy D. G. et al. (2004). Hereditary early-onset Parkinson's disease caused by mutations in PINK1. *Science* 304, 1158-1160. 10.1126/science.1096284 15087508

[BIO022186C27] Virág L. and Szabó C. (2002). The therapeutic potential of poly(ADP-ribose) polymerase inhibitors. *Pharmacol. Rev.* 54, 375-429. 10.1124/pr.54.3.375 12223530

[BIO022186C28] Virág L., Salzman A. L. and Szabo C. (1998). Poly(ADP-ribose) synthetase activation mediates mitochondrial injury during oxidant-induced cell death. *J. Immunol.* 161, 3753-3759. 9759901

[BIO022186C29] Vos M., Esposito G., Edirisinghe J. N., Vilain S., Haddad D. M., Slabbaert J. R., Van Meensel S., Schaap O., De Strooper B., Meganathan R. et al. (2012). Vitamin K2 is a mitochondrial electron carrier that rescues pink1 deficiency. *Science* 336, 1306-1310. 10.1126/science.1218632 22582012

[BIO022186C30] Wang X.-G., Wang Z.-Q., Tong W.-M. and Shen Y. (2007). PARP1 Val762Ala polymorphism reduces enzymatic activity. *Biochem. Biophys. Res. Commun.* 354, 122-126. 10.1016/j.bbrc.2006.12.162 17214964

[BIO022186C31] Wexler E. J. (2008). *Photoshop CS3 Extended for Biomedical Research*. Ventura: Lynda.com, Inc.

[BIO022186C32] Whitworth A. J., Theodore D. A., Greene J. C., Benes H., Wes P. D. and Pallanck L. J. (2005). Increased glutathione S-transferase activity rescues dopaminergic neuron loss in a *Drosophila* model of Parkinson's disease. *Proc. Natl. Acad. Sci. USA* 102, 8024-8029. 10.1073/pnas.0501078102 15911761 PMC1142368

[BIO022186C33] Zhang P. and Spradling A. C. (1994). Insertional mutagenesis of *Drosophila* heterochromatin with single P elements. *Proc. Natl. Acad. Sci. USA* 91, 3539-3543. 10.1073/pnas.91.9.3539 8170943 PMC43615

[BIO022186C34] Zuo L. and Motherwell M. S. (2013). The impact of reactive oxygen species and genetic mitochondrial mutations in Parkinson's disease. *Gene* 532, 18-23. 10.1016/j.gene.2013.07.085 23954870

